# Recognition of 5-Hydroxymethylcytosine by the Uhrf1 SRA Domain

**DOI:** 10.1371/journal.pone.0021306

**Published:** 2011-06-22

**Authors:** Carina Frauer, Thomas Hoffmann, Sebastian Bultmann, Valentina Casa, M. Cristina Cardoso, Iris Antes, Heinrich Leonhardt

**Affiliations:** 1 Department of Biology II, Ludwig Maximilians University Munich, Planegg-Martinsried, Germany; 2 Department of Life Sciences, Technical University Munich, Freising-Weihenstephan, Germany; 3 Department of Biology, Technical University Darmstadt, Darmstadt, Germany; 4 Center for Integrated Protein Science Munich (CIPSM), Munich, Germany; New England Biolabs, Inc., United States of America

## Abstract

Recent discovery of 5-hydroxymethylcytosine (5hmC) in genomic DNA raises the question how this sixth base is recognized by cellular proteins. In contrast to the methyl-CpG binding domain (MBD) of MeCP2, we found that the SRA domain of Uhrf1, an essential factor in DNA maintenance methylation, binds 5hmC and 5-methylcytosine containing substrates with similar affinity. Based on the co-crystal structure, we performed molecular dynamics simulations of the SRA:DNA complex with the flipped cytosine base carrying either of these epigenetic modifications. Our data indicate that the SRA binding pocket can accommodate 5hmC and stabilizes the flipped base by hydrogen bond formation with the hydroxyl group.

## Introduction

DNA methylation is an epigenetic modification that is well known to control eukaryotic gene expression [1], [2]. In fact, methylation of regulatory sequences often correlates with a transcriptionally silent state. DNA methylation in mammals occurs as 5-methylcytosine (5mC) within CpG dinucleotides and is catalyzed by a family of DNA methyltransferases (Dnmts) [3]. Dnmt members are distinguished by their function; while the *de novo* methyltransferases Dnmt3a and Dnmt3b establish methylation patterns during development and cellular differentiation [4], [5], the *maintenance* methyltransferase Dnmt1 copies these patterns during DNA replication [6], [7], [8]. Although DNA methylation per se can prevent binding of transcriptional regulators [9], the main mechanism by which transcriptional repression is achieved appears to involve 5mC binding proteins (MBPs). MBPs specifically recognize methylation marks and consequently stabilize silent chromatin states by recruitment of histone modifying enzymes and chromatin remodeling factors [10].

There are three families of MBPs known to date: the methyl-CpG binding domain (MBD) family, the Uhrf family and the Kaiso protein family. In contrast to the members of the MBD and Kaiso families that specifically recognize fully methylated CpG sites, Uhrf1, the best characterized member of the Uhrf family, preferentially binds hemimethylated DNA, the substrate of maintenance methylation [11], [12], [13], [14]. Notably, crystal structures of the DNA binding domains of MeCP2 and Uhrf1 in complex with DNA revealed striking differences: whereas the MeCP2 MBD recognizes methylated CpG sites based on hydration of the DNA major groove, the Uhrf1 (Set and Ring associated) SRA domain uses a base-flipping mechanism to bind DNA containing hemimethylated CpG sites [11], [12], [14], [15]. Interestingly, Uhrf1 recently emerged as essential cofactor for maintenance methylation potentially by recruiting Dnmt1 to its target sites [13], [16], [17].

In addition to 5mC, genomic DNA has been recently shown to contain 5-hydroxy-methylcytosine (5hmC), which results from oxidation of 5mC catalyzed by Tet proteins [18], [19], [20]. This new modification has been implicated in DNA demethylation, either passively as 5hmC containing DNA is not a substrate for Dnmt1 [21], or actively by so far unknown mechanisms. The central questions remain which proteins recognize 5hmC modified DNA and whether 5hmC has a direct role in gene regulation similar to its analog 5mC.

In this study, we characterized the 5mC/5hmC DNA binding properties of two representative 5mC binding protein domains, the MBD of MeCP2 and the SRA domain of Uhrf1. We found that in contrast to the MBD, the SRA domain binds hydroxymethylated DNA substrates with similar affinity as methylated substrates. We investigated the binding mode and energies of Uhrf1 to DNA substrates containing 5mC and 5hmC using molecular dynamics simulations of the respective SRA:DNA complexes.

## Results

### Uhrf1 binds DNA substrates containing hydroxymethylated CpG sites

**Figure 1:**
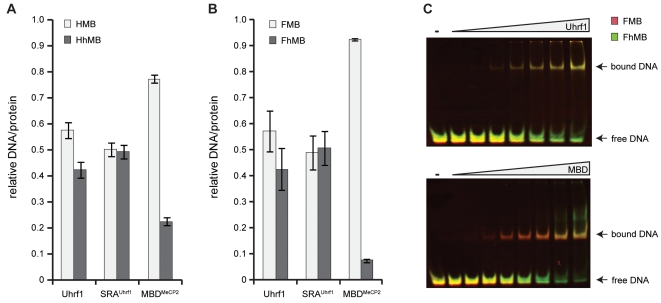
DNA binding specificity of 5-methylcytosine binding proteins. (**A+B**) Relative DNA/protein ratios of Uhrf1, its SRA domain (SRA^Uhrf1^) and the MBD of MeCP2 (MBD^MeCP2^) with two differentially labeled DNA substrates in direct competition. (A) Binding to DNA substrates containing a hemimethylated or hemihydroxymethylated CpG site (HMB versus HhMB, respectively). (B) Binding to DNA substrates containing a fully methylated or fully hydroxymethylated CpG site (FMB versus FhMB, respectively). Results are shown as means of three independent experiments with standard deviation error bars. Note that MBD^MeCP2^ preferentially binds to FMB, whereas the Uhrf1 constructs do not discriminate between FMB and FhMB. (**C**) Electrophoretic mobility shift assays were performed with Uhrf1 or MBD^MeCP2^ and equimolar amounts of FMB (red) and FhMB (green) in competition. The overlay of the two substrate channels reveals simultaneous shifting of both DNA substrates with Uhrf1, whereas with MBD^MeCP2^ the FMB substrate shifts at a lower protein concentration than the FhMB substrate, confirming differential binding.

Using a newly established DNA binding assay [22], [23] as well as electrophoretic mobility shift assays, we investigated the DNA binding activity of Uhrf1, its SRA domain (SRA^Uhrf1^) and the MBD of MeCP2 (MBD^MeCP2^) to methylated and hydroxymethylated DNA in direct competition (Figure 1, Supplementary Figure S1; note that all supplementary information can also be found in the Combined Supporting Information File S1). We found that the Uhrf1 constructs bind 5mC and 5hmC containing substrates with similar affinities independent of whether one or both cytosine residues of the palindromic CpG site were modified. Control experiments performed with hemimethylated DNA in competition with either unmethylated substrates or substrates containing no CpG site showed that the observed binding activity to methylated and hydroxymethylated DNA is indeed specific (Supplementary Figure S2). In stark contrast to Uhrf1, we found that MBD^MeCP2^ clearly discriminates between methylation and hydroxymethylation, which is in accordance with previous reports [21], [24].

### Molecular dynamics simulations of SRA:DNA complexes with 5mC and 5hmC

**Figure 2:**
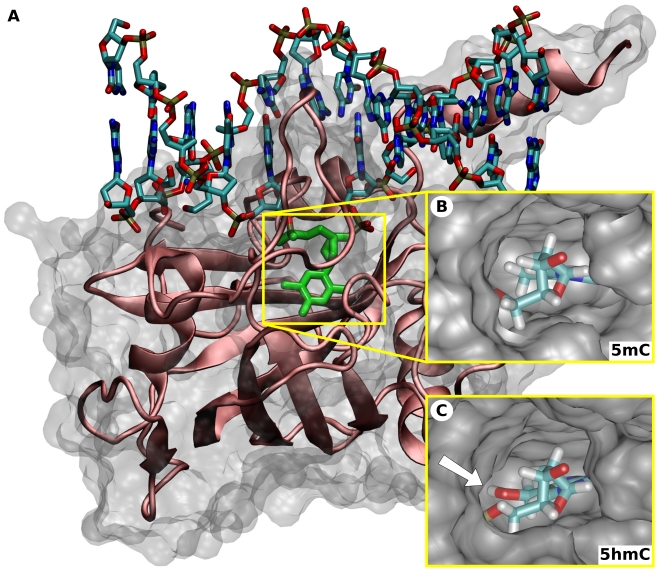
Structure of the Uhrf1 SRA domain in complex with hemimethylated and hemihydroxymethylated DNA. (**A**) Experimental structure of the Uhrf1 SRA domain in complex with hemimethylated DNA (PDB-ID:3fde, [14]). The protein is shown in cartoon and the DNA in licorice representation. The 5mC nucleotide is highlighted in green. Note that the 5mC residue is flipped out of the DNA double helix. (**B+C**) Models of the SRA binding pocket with bound 5mC (B) and 5hmC (C) serving as starting points for the molecular dynamics simulations. The location of the hydroxyl group in the 5hmC complex is highlighted by the white arrow. The view is from the top of the binding site (DNA backbone) and rotated by 90 degrees compared to (A).

**Figure 3:**
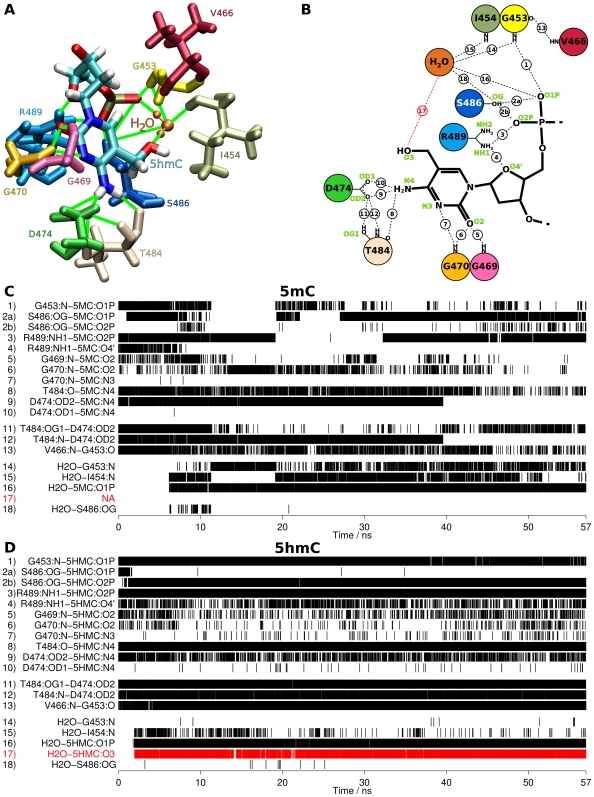
Molecular dynamics simulations of the SRA domain in complex with 5mC and 5hmC containing DNA. (**A+B**) Three and two-dimensional schematic drawings summarizing the hydrogen bond networks between the nucleotides, the SRA binding pocket, and a conserved water molecule during the simulations. The numbers in (B) correspond to the numbering in (C+D). (**C+D**) Hydrogen bond occurrences during the molecular dynamics simulations of the SRA domain in complex with either 5mC (C) or 5hmC containing DNA (D). Each vertical line represents a single observed hydrogen bond. The hydrogen bond between 5hmC and the conserved water is highlighted in red.

To investigate the binding mode of the SRA domain to DNA containing 5mC or 5hmC, we performed molecular dynamics simulations for both SRA:DNA complexes. Consistent with the *in vitro* DNA binding data, modeling of an additional hydroxyl group into the complex structure of the Uhrf1 SRA domain with DNA containing hemimethylated CpG sites revealed no spatial constraints for accommodation of the flipped 5hmC nucleotide within the binding pocket (Figure 2). Based on these initial models of the bound conformation, we performed molecular dynamics simulations for a time interval of 57 ns and monitored the RMSD and RMSF values (Supplementary Figures S3 and S4). In both systems equilibrium was reached after 20 to 30 ns. To assure evaluation of equilibrated systems, we continued the equilibrium simulations for another 27 ns and used only the last 10 ns for subsequent interaction energy analysis [25]. To evaluate the stability of the flipped nucleotides within the binding site, we monitored the occurrence and stability of all hydrogen bonds in the vicinity of the binding site with respect to the progress of the simulations (Figure 3).

Before starting the simulations, all water molecules from the X-ray structure were removed and new water molecules were placed by the setup solvation algorithm of NAMD [26]. Therefore, no water molecules were present in the vicinity of the flipped nucleotides at the beginning of the simulations. Interestingly, in both simulations, water molecules from the water-filled simulation box moved into the nucleotide binding site within the first couple of nanoseconds (Figures 3C and 3D, hydrogen bonds 14 to 18). During the remainder of the simulation time, one water molecule was stabilized within the binding site by formation of distinct hydrogen bonds with protein and DNA. Notably, the position of this water molecule in the 5mC complex corresponds to that of a conserved water molecule in the experimental structure (Supplementary Figure S5), confirming the stability and accuracy of our simulations.

**Figure 4:**
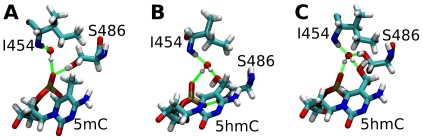
Hydrogen bond networks stabilizing 5mC and 5hmC within the SRA binding pocket. (**A**) SRA complex with DNA containing 5mC. (**B+C**) SRA complex with DNA containing 5hmC. In the 5hmC complex, the water molecule stably interacts with the hydroxyl group of the nucleotide, but two alternative conformations of the SRA binding pocket exist depending on the ion concentration. In the absence of salt, binding involves an interaction of the S486 residue with the phosphate group of the flipped nucleotide (B), whereas in the presence of 0.5 M NaCl, residue S486 interacts with the conserved water molecule (C).

Despite the presence of a conserved water molecule in the binding pockets of both complexes, the corresponding hydrogen bond networks showed interesting differences. In the 5mC complex, this water molecule forms hydrogen bonds with the phosphodiester group of the methylated nucleotide as well as with the SRA residues I454 and G453, thereby bridging the DNA backbone:protein interaction (Figure 3A–C, hydrogen bonds 14–16, Figure 4A). Furthermore, direct hydrogen bonds between the 5mC DNA backbone and the protein are formed involving residues G453, S486, and R489 (hydrogen bonds 1–4).

The hydrogen bond network of the 5hmC complex is more stable compared to the 5mC complex (Figure 3D, compare with 3C). Most prominently, one additional and very stable hydrogen bond is formed between the conserved water molecule and the hydroxyl group of the 5hmC nucleotide (hydrogen bond 17). This interaction seems to specifically stabilize the hydrogen bonding network between the DNA backbone and the binding pocket residues G453, S486, and R489 (hydrogen bonds 1–4). Interestingly, these hydrogen bonds have been previously identified to be important for DNA binding [14] and possibly stabilize the flipped conformation of the nucleotide within the binding site. In addition, the hydrogen bond network within the protein involving residues V466 and G453 as well as residues T484 and D474 is stabilized in the 5hmC complex (hydrogen bonds 11–13).

Since water dynamics and to some extent also DNA dynamics can depend on the ion concentration parameters used in the molecular dynamics simulation, we performed a second simulation of the 5hmC complex with a higher ion concentration (Supplementary Figure S6). Consistent to the first simulation with 5hmC, we observed the same overall water dynamics and hydrogen bonding patterns including hydrogen bond formation between the hydroxyl group of the 5hmC nucleotide and the conserved water molecule within the SRA structure. Notably, the stable hydrogen bonding between protein residue S486 and the DNA backbone in the first simulation (hydrogen bonds 2a and 2b) seems to be replaced by a stable hydrogen bond of S486 with the water molecule in the second simulation (hydrogen bond 18), indicating two alternative interaction patterns for the S486 residue in the 5hmC complex (Figures 4B and 4C, compare Figure 3D and Supplementary Figure S6B). In conclusion, these data suggest that stable, water bridged hydrogen bond formation of the hydroxyl group of the flipped 5hmC nucleotide with its surrounding occurs in and stabilizes this DNA:SRA complex.

### Similar interaction energies for SRA complexes with 5mC and 5hmC containing DNA

To estimate the binding affinity between the Uhrf1 SRA domain and DNA containing either 5mC or 5hmC, we calculated the respective interaction energies using the linear interaction energy (LIE) approach [25]. To exclude energy contributions due to base-flipping when comparing the interaction of the DNA with the protein (bound state) or with the solvent (unbound state), we simulated the DNA in a flipped state in both cases. We determined the difference between the binding energies of the two complexes (ΔΔG = ΔG_5mC_−ΔG_5hmC_). We included either i) the whole DNA and SRA structure (ΔΔG = −7.94 kcal/mol) or ii) the flipped nucleotide with its five neighboring nucleotides and the binding pocket of the protein, defined as all residues within a distance of 15 Å from the nucleotide in the starting conformation (ΔΔG = −6.65 kcal/mol). These values suggest that the slight difference in binding affinity is predominantly due to interaction of the flipped nucleotide with the proximal protein residues that form the binding site. Considering the estimated uncertainty of about 3–4 kcal/mol in our calculations, these values indicate that both 5mC and 5hmC containing DNA substrates bind with very similar affinity to the SRA domain of Uhrf1.

## Discussion

In summary, we observed fundamentally different binding specificities for the DNA binding domains of representative 5mC binding proteins. Hydroxylation of 5mC clearly interferes with DNA binding by the MBD of MeCP2 and might prevent subsequent establishment of repressive chromatin structures in a cellular context, thereby changing the cellular interpretation of an epigenetic modification. Notably, MeCP2 expression is highest in brain tissues where also 5hmC levels are highest [18], [27], [28]. In stark contrast, Uhrf1, a key factor in maintenance methylation, recognizes 5hmC as well as 5mC. The results of our molecular dynamics simulations provide a structural explanation for recognition of 5hmC. Interestingly, the flipped 5hmC base not only fits into the binding pocket of the Uhrf1 SRA domain, but is specifically stabilized by hydrogen bond formation involving the 5hmC hydroxyl group. This interaction is bridged by a conserved water molecule present within the SRA binding pocket and seems to stabilize the overall hydrogen bond network of the 5hmC complex. Also in the 5mC complex a conserved water molecule is found in the vicinity of the flipped cytosine, which in this case, however, only interacts with the SRA domain and the backbone of the DNA and not with the flipped nucleotide itself.

The specific binding of Uhrf1 to 5hmC containing DNA was clearly unexpected and puts the existing hypothesis on Uhrf1 function into a new perspective. Knock-out studies in mouse embryonic stem cells and embryos revealed that Uhrf1 is essential for maintenance DNA methylation by Dnmt1 [17]. Based on the specific binding of Uhrf1 to hemimethylated CpG sites and its interaction with Dnmt1, Uhrf1 was suggested to operate by recruiting Dnmt1 to its target sites [11], [12], [13], [14], [17]. Recent studies suggested a role of hydroxymethylation in passive [21] and/or active [29], [30], [31] DNA demethylation. The binding of Uhrf1 to hydroxymethylated DNA reported in this study now raises the question how Uhrf1 contributes to change or maintenance of methylation *in vivo*. In this context it should also be noted that the preferential binding of Uhrf1 to hemimethylated DNA is relatively weak, especially if compared to the intrinsic preference of Dnmt1 for methylation of these substrates [22], [23]. Moreover, multiple interactions of Uhrf1 with repressive histone tail modifications [23] as well as other heterochromatin associated proteins [32], [33] seem to be required for the specific localization and targeting of Uhrf1 *in vivo*. Together, these data strongly argue for a more complex mechanism of Uhrf1 function in living cells and emphasize the need for further studies to understand the pivotal role of Uhrf1 in the establishment, maintenance and change of genome-wide methylation patterns.

Using a combination of *in vitro* and *in silico* studies, we clearly demonstrate that Uhrf1 can bind 5hmC containing DNA. It still remains elusive whether or in which specific context Uhrf1 binds 5hmC modified DNA substrates in living cells. Uhrf1 binding to 5hmC and possible functional consequences *in vivo* are likely to depend on additional interacting factors. Comparison of genome-wide Uhrf1 ChIP profiles with 5mC and 5hmC distribution should help to clarify the interactions and functions of Uhrf1 *in vivo*. Finally, it is interesting to note that Uhrf1 is the only base-flipping protein with so far unknown catalytic function on DNA. The direct interaction of a water molecule with the hydroxyl group of 5hmC within the SRA binding pocket might possibly point towards a role of Uhrf1 in the further modification of this sixth DNA base. In conclusion, our study provides new perspectives on the cellular interpretation and possible further metabolism of this new epigenetic DNA modification.

## Materials and Methods

### Expression constructs, cell culture and transfection

Mammalian expression constructs for enhanced green fluorescent protein (GFP), Uhrf1 (GFP-Uhrf1), the SRA domain of Uhrf1 (GFP-SRA^Uhrf1^) and the MBD of MeCP2 (MBD^MeCP2^-YFP) were described previously [22], [23], [34]. Note that all constructs encode fusion proteins of either GFP or yellow fluorescent protein (YFP). HEK293T cells [35] were cultured in DMEM supplemented with 50 µg/ml gentamicin and 10% fetal calf serum. For expression of GFP/YFP fusion proteins, HEK293T cells were transfected with the corresponding expression constructs using polyethylenimine (Sigma).

### DNA substrate preparation

Fluorescently labeled DNA substrates were prepared by mixing two HPLC-purified DNA oligonucleotides (IBA GmbH, Supplementary Tables S1 and S2) in equimolar amounts, denaturation for 30 sec at 92°C and slow cool-down to 25°C allowing hybridization. After purification by 15% non-denaturing PAGE, DNA substrates were resuspended in binding buffer (20 mM TrisHCl pH 7.5, 150 mM NaCl, 1 mM EDTA, 1 mM DTT).

### Pull-down DNA binding assay

*In vitro* DNA binding assays were performed as described previously [22], [23]. In brief, GFP/YFP fusions were purified from HEK293T extracts using the GFP-Trap® (ChromoTek GmbH) and incubated with two differentially labeled DNA substrates at a final concentration of 200 nM DNA/50–100 nM immobilized protein for 45 min at room temperature in binding buffer. After removal of unbound substrate, the amounts of protein and DNA were determined by fluorescence intensity measurements with a Tecan Infinite M1000 plate reader. Binding ratios were calculated dividing the concentration of bound DNA substrate by the concentration of GFP/YFP fusion on the beads, corrected by values from a control experiment using DNA substrates of the same sequence but with different fluorescent labels, and normalized by the total amount of bound DNA.

### Electrophoretic mobility shift assay

For competitive electrophoretic mobility shift assays, equimolar amounts of two differentially labeled DNA substrates (250 nM each) were incubated with increasing amounts of GFP/YFP fusion protein (Supplementary Figure S1), subjected to 6% non-denaturing PAGE and analyzed with a Typhoon scanner (GE Healthcare), which allowed separate detection of DNA substrates and protein by ATTO labels and GFP tag, respectively, using the following laser/filter settings: 532 nm/580 nm (ATTO550), 633 nm/none (ATTO700), 488 nm/520 nm (GFP/YFP).

### Molecular dynamics simulations

Molecular dynamics simulations were performed based on the X-ray structure of the Uhrf1 SRA domain with the PDB identifier 3FDE [14], using the program NAMD 2.7b1 [26] and the CHARMM22/27 force field [36], [37]. Binding free energies were estimated using the Linear Interaction Energy (LIE) model [25].

After energy minimization of 50,000 steps, one hydrogen atom of the methyl group of the protein-bound 5-methylcytosine (5mC) residue was substituted by a hydroxyl group using the tool psfgen. CHARMM22 force field parameters were available for 5mC (patch: PRES 5MC2), but not for 5-hydroxymethylcytosine (5hmC). Therefore, a new 5hmC residue was created based on the 5mC parameters and topology. For this purpose, one hydrogen atom of the 5mC methyl group was exchanged by a hydroxyl group. The charges of the hydroxyl group were subsequently set to charges of the hydroxyl group of a serine residue according to the CHARMM27; the charges of the CH_2_ group were adjusted accordingly (Supplementary Table S3). After solvation, the 5mC and 5hmC structures were further energy minimized for 50,000 steps. For each structure, two simulations were performed, in which the charges were either neutralized or a salt concentration of 0.5 M was used.

Each simulation was performed using periodic boundary conditions and particle-mesh-ewald summation [38] for long range non-bonded interactions. The non-bonded cutoff was set to 14 Å with a switching/shifting distance of 12 Å. A stepsize of 1 fs was chosen. The systems were heated from 0 to 200 K for 160 ps under constant volume. Harmonic restraints (1000 kcal mol^−1^ nm^−2^) were applied to all atoms of the complex. The heat up was continued without harmonic restraints from 200 to 300 K for 80 ps under constant pressure conditions, using a Nose-Hoover barostat [39], [40] with a target pressure of 1.01325 bar, an oscillation time scale of 100 fs, and a damping time scale of 50 fs. The temperature was maintained by Langevin dynamics using a damping coefficient of 5/ps. The temperature bath was not coupled to hydrogen atoms. After the heat up procedure, the simulations were continued for 57 ns. During the simulations, all bond lengths were constrained to ideal values using the Shake algorithm [41], [42].

For analysis of the simulation results, all hydrogen bonds formed by the flipped nucleotides and the binding site were identified and monitored throughout the simulations and the occurrence of water molecules in and around the binding site was monitored every 5 ps. In order to estimate the difference in the binding free energy of the two nucleotides, we performed three further simulations in which the protein and the two DNA molecules were simulated separately using the conditions described above. To keep the DNA in the flipped state, we additionally applied harmonic restraints to the whole DNA backbone (atom names: C4′, P, O1P, O2P, O5′, C5′, C3′, O3′). The solvated single protein was simulated for 34 ns and the separated DNA molecules were simulated for 20 ns.

To estimate the binding affinity of the two DNA molecules to the protein, we estimated the binding free energy according to the Linear Interaction Energy (LIE) model [25]:
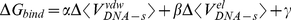
(1)

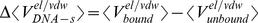
(2)


In this approach the binding free energy is approximated by the difference between the interaction energies Δ*V^el^* and Δ*V^vdw^* of the ligand in the protein-ligand complex (bound state) and in solution (unbound state). The <> denotes the average values obtained from the simulation trajectories. According to the linear response approximation the weights α and β were set to 1 and 0.5, respectively. We calculated the DNA-(protein+solvent) (bound state) and the DNA-solvent (free state) interaction energies from the trajectories of the DNA/SRA and the DNA/solvent simulations, using the average energy over the last 10 ns.

## Supporting Information

Figure S1 **Electrophoretic mobility shift assays with methylated and hydroxymethylated DNA substrates.** Increasing amounts of Uhrf1, its SRA domain (SRA^Uhrf1^) or the MBD domain of MeCP2 (MBD^MeCP2^) were incubated with two differentially ATTO-labeled DNA substrates, which contain either one central fully methylated or fully hydroxymethylated CpG site (FMB-ATTO700 or FhMB-ATTO550, respectively), in direct competition. Samples were subjected to 6% non-denaturing PAGE and analyzed with a Typhoon scanner (GE Healthcare). The first, second and third columns show the scans for GFP/YFP, ATTO700 and ATTO550 fluorescence, respectively. The overlay of the two ATTO channels is shown in the fourth column (FMB: red, FhMB:green).
(PDF)

Figure S2 **DNA binding specificity of Uhrf1.** Relative DNA/Uhrf1 ratios are shown for two differentially labeled fluorescent DNA substrates in direct competition. (A) Binding of Uhrf1 to DNA substrates containing no CpG site or one central hemimethylated CpG site (noCGB versus HMB, respectively). (B) Binding of Uhrf1 to DNA substrates containing one central un- or hemimethylated CpG site (UMB versus HMB, respectively). Results are shown as means of three independent experiments with standard deviation error bars. DNA substrates were prepared by hybridization as described in the main text, except for noCGB, which was prepared by primer extension as described previously [22]. See Supplementary Tables S1 and S2 for DNA oligonucleotide sequences and purification grade of the used substrates.
(PDF)

Figure S3 **Atom-positional root-mean-square deviation of the protein and DNA backbone atoms during the simulations.** The terminal DNA and protein residues were excluded from the calculations in the “subset” sets (red and black lines).
(PDF)

Figure S4 **Atom-positional root-mean-square fluctuations of the protein (A, C) and both DNA strands (B, D) during two simulation periods.** Note that both structures show the same flexibility pattern during both simulation periods and are overall stable during both periods. This is in agreement with the RMSD data in Figure S3, which shows that equilibration is reached after 30 ns of simulation time.
(PDF)

Figure S5 **Superposition of the equilibrated 5mC structure after simulation (atom-name specific coloring) and the crystal structure (PDB-ID:3fde** [**14**] **, green).** The 5mC nucleotide, the residue I454 of the SRA binding pocket and the conserved water molecule are shown. Note that the distance between the oxygen atoms of the conserved water molecules in the two structures is only 1.1 Å.
(PDF)

Figure S6 **Molecular dynamics simulations of the Uhrf1 SRA domain in complex with 5mC (A) and 5hmC (B) containing DNA in 0.5 M NaCl.** Hydrogen bond occurrences during the simulation of the SRA:DNA complex using a concentration of 0.5 M NaCl.
(PDF)

Table S1 **Sequences of DNA oligonucleotides used for preparation of double stranded fluorescent DNA substrates.** M: 5-methylcytosine. X: 5-hydroxymethylcytosine.
(PDF)

Table S2 **DNA substrates used for the DNA binding assays.**
(PDF)

Table S3 **Residue Topology File and parameters used for the 5hmC residue during the simulations.**
(PDF)

File S1 **Combined supporting figures and tables.**
(PDF)
