# Supplementary material for: Recognition of 5-Hydroxymethylcytosine by the Uhrf1 SRA Domain
Source: PLoS One. 2011 Jun 22;6(6):e21306. doi: 10.1371/journal.pone.0021306 (PMC3120858; doi:10.1371/journal.pone.0021306)
Supplement: Figure S1 — Electrophoretic mobility shift assays with methylated and hydroxymethylated DNA substrates. Increasing amounts of Uhrf1, its SRA domain (SRAUhrf1) or the MBD domain of MeCP2 (MBDMeCP2) were incubated with two differentially ATTO-labeled DNA substrates, which contain either one central fully methylated or fully hydroxymethylated CpG site (FMB-ATTO700 or FhMB-ATTO550, respectively), in direct competition. Samples were subjected to 6% non-denaturing PAGE and analyzed with a Typhoon scanner (GE Healthcare). The first, second and third columns show the scans for GFP/YFP, ATTO700 and ATTO550 fluorescence, respectively. The overlay of the two ATTO channels is shown in the fourth column (FMB: red, FhMB:green). (PDF) [file pone.0021306.s001.pdf]

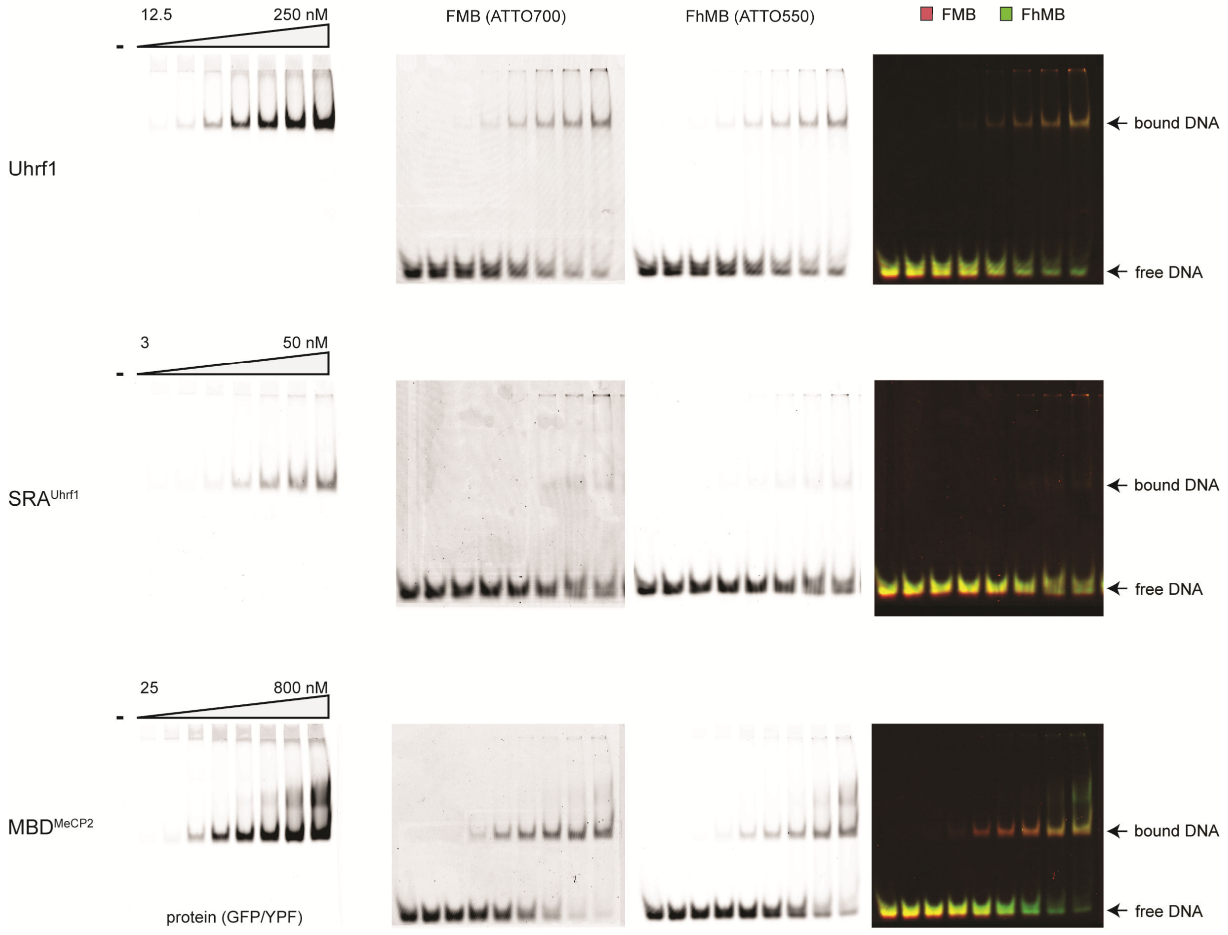

Supplementary Figure S1. Electrophoretic mobility shift assays with methylated and hydroxymethylated DNA substrates. Increasing amounts of Uhrf1, its SRA domain (SRA^{Uhrf1}) or the MBD domain of MeCP2 (MBD^{MeCP2}) were incubated with two differentially ATTO-labeled DNA substrates, which contain either one central fully methylated or fully hydroxymethylated CpG site (FMB-ATTO700 or FhMB-ATTO550, respectively), in direct competition. Samples were subjected to 6 % non-denaturing PAGE and analyzed with a Typhoon scanner (GE Healthcare). The first, second and third columns show the scans for GFP/YFP, ATTO700 and ATTO550 fluorescence, respectively. The overlay of the two ATTO channels is shown in the fourth column (FMB: red, FhMB:green).
