# Supplementary material for: Recognition of 5-Hydroxymethylcytosine by the Uhrf1 SRA Domain
Source: PLoS One. 2011 Jun 22;6(6):e21306. doi: 10.1371/journal.pone.0021306 (PMC3120858; doi:10.1371/journal.pone.0021306)
Supplement: Figure S2 — DNA binding specificity of Uhrf1. Relative DNA/Uhrf1 ratios are shown for two differentially labeled fluorescent DNA substrates in direct competition. (A) Binding of Uhrf1 to DNA substrates containing no CpG site or one central hemimethylated CpG site (noCGB versus HMB, respectively). (B) Binding of Uhrf1 to DNA substrates containing one central un- or hemimethylated CpG site (UMB versus HMB, respectively). Results are shown as means of three independent experiments with standard deviation error bars. DNA substrates were prepared by hybridization as described in the main text, except for noCGB, which was prepared by primer extension as described previously [22]. See Supplementary Tables S1 and S2 for DNA oligonucleotide sequences and purification grade of the used substrates. (PDF) [file pone.0021306.s002.pdf]

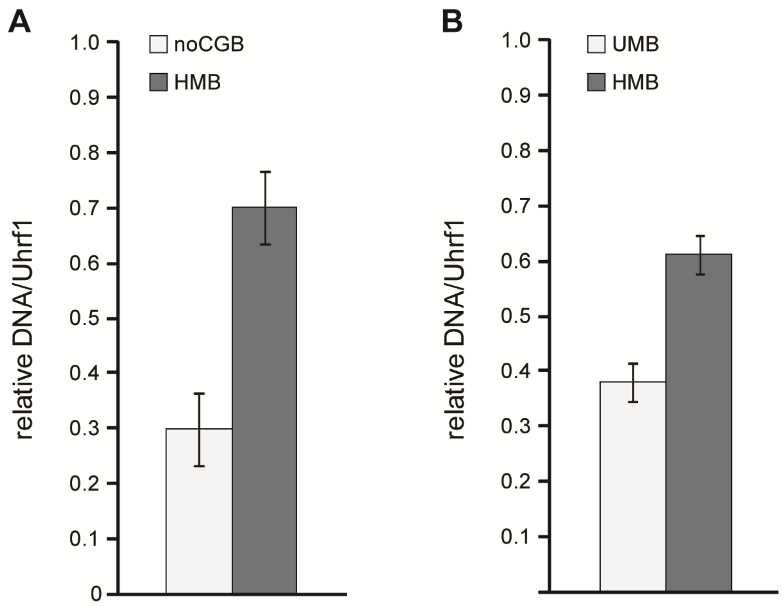

Supplementary Figure S2. DNA binding specificity of Uhrf1. Relative DNA/Uhrf1 ratios are shown for two differentially labeled fluorescent DNA substrates in direct competition. (A) Binding of Uhrf1 to DNA substrates containing no CpG site or one central hemimethylated CpG site (noCGB versus HMB, respectively). (B) Binding of Uhrf1 to DNA substrates containing one central un- or hemimethylated CpG site (UMB versus HMB, respectively). Results are shown as means of three independent experiments with standard deviation error bars. DNA substrates were prepared by hybridization as described in the main text, except for noCGB, which was prepared by primer extension as described previously [1]. See Supplementary Tables 1 and 2 for DNA oligonucleotide sequences and purification grade of the used substrates.

1. Frauer C, Leonhardt H (2009) A versatile non-radioactive assay for DNA methyltransferase activity and DNA binding. Nucleic Acids Res 37: e22.
