# Supplementary material for: Recognition of 5-Hydroxymethylcytosine by the Uhrf1 SRA Domain
Source: PLoS One. 2011 Jun 22;6(6):e21306. doi: 10.1371/journal.pone.0021306 (PMC3120858; doi:10.1371/journal.pone.0021306)
Supplement: Figure S3 — Atom-positional root-mean-square deviation of the protein and DNA backbone atoms during the simulations. The terminal DNA and protein residues were excluded from the calculations in the “subset” sets (red and black lines). (PDF) [file pone.0021306.s003.pdf]

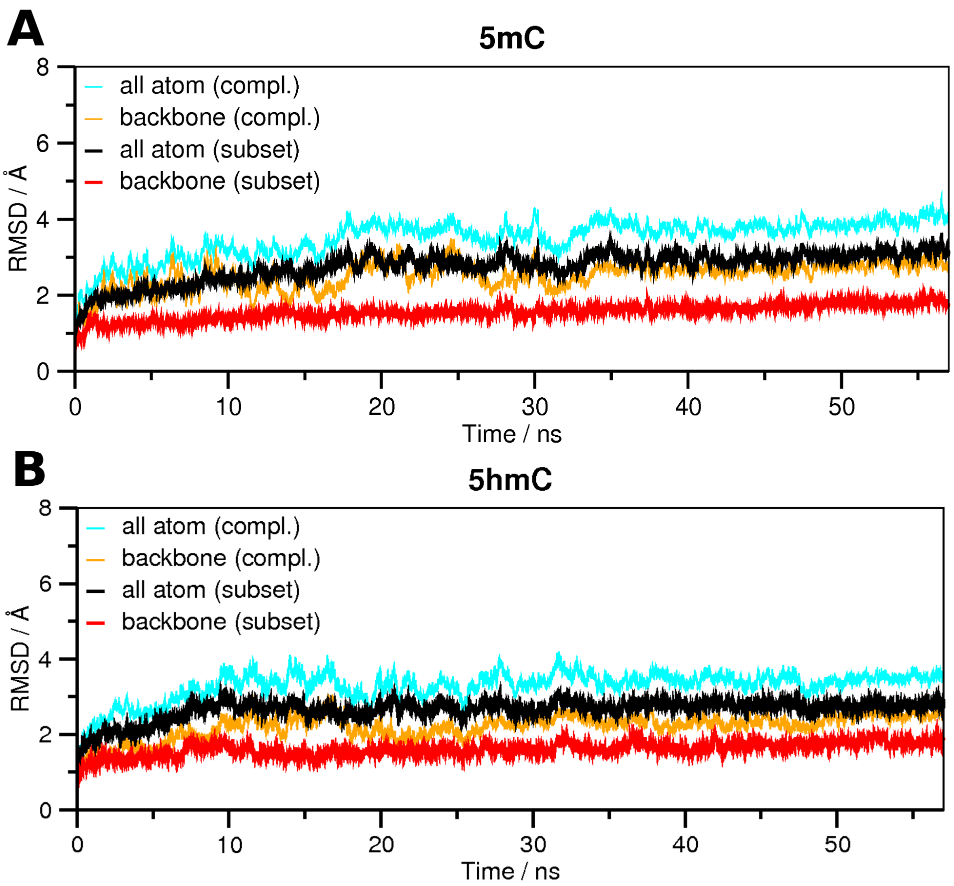

Supplementary Figure S3. Atom-positional root-mean-square deviation of the protein and DNA backbone atoms during the simulations. The terminal DNA and protein residues were excluded from the calculations in the "subset" sets (red and black lines).
