# Supplementary material for: Recognition of 5-Hydroxymethylcytosine by the Uhrf1 SRA Domain
Source: PLoS One. 2011 Jun 22;6(6):e21306. doi: 10.1371/journal.pone.0021306 (PMC3120858; doi:10.1371/journal.pone.0021306)
Supplement: Figure S4 — Atom-positional root-mean-square fluctuations of the protein (A, C) and both DNA strands (B, D) during two simulation periods. Note that both structures show the same flexibility pattern during both simulation periods and are overall stable during both periods. This is in agreement with the RMSD data in Figure S3, which shows that equilibration is reached after 30 ns of simulation time. (PDF) [file pone.0021306.s004.pdf]

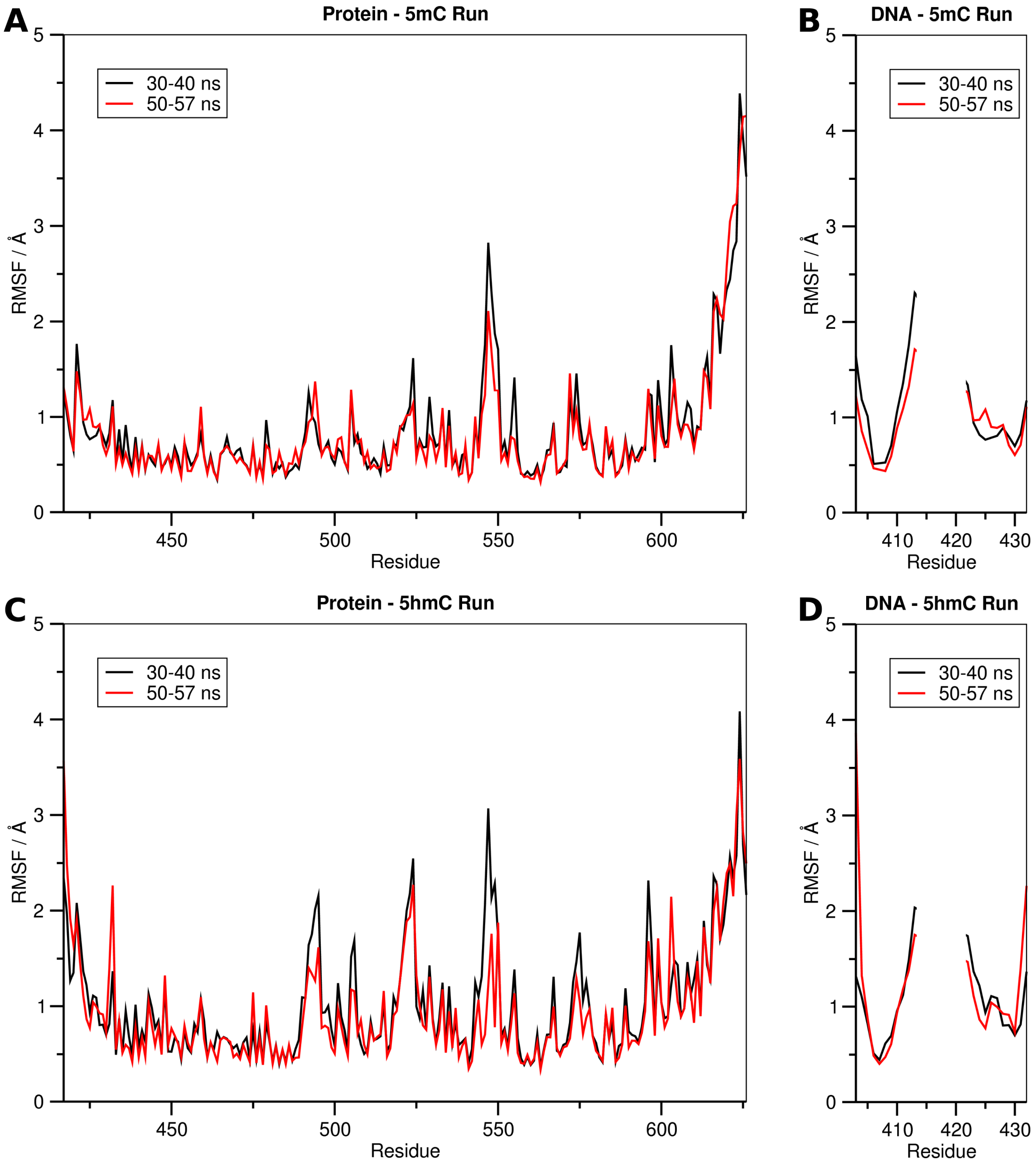

Supplementary Figure S4. Atom-positional root-mean-square fluctuations of the protein (A, C) and both DNA strands (B, D) during two simulation periods. Note that both structures show the same flexibility pattern during both simulation periods and are overall stable during both periods. This is in agreement with the RMSD data in Figure S3, which shows that equilibration is reached after 30 ns of simulation time.
