# Supplementary material for: Recognition of 5-Hydroxymethylcytosine by the Uhrf1 SRA Domain
Source: PLoS One. 2011 Jun 22;6(6):e21306. doi: 10.1371/journal.pone.0021306 (PMC3120858; doi:10.1371/journal.pone.0021306)
Supplement: Figure S5 — Superposition of the equilibrated 5mC structure after simulation (atom-name specific coloring) and the crystal structure (PDB-ID:3fde [14] , green). The 5mC nucleotide, the residue I454 of the SRA binding pocket and the conserved water molecule are shown. Note that the distance between the oxygen atoms of the conserved water molecules in the two structures is only 1.1 Å. (PDF) [file pone.0021306.s005.pdf]

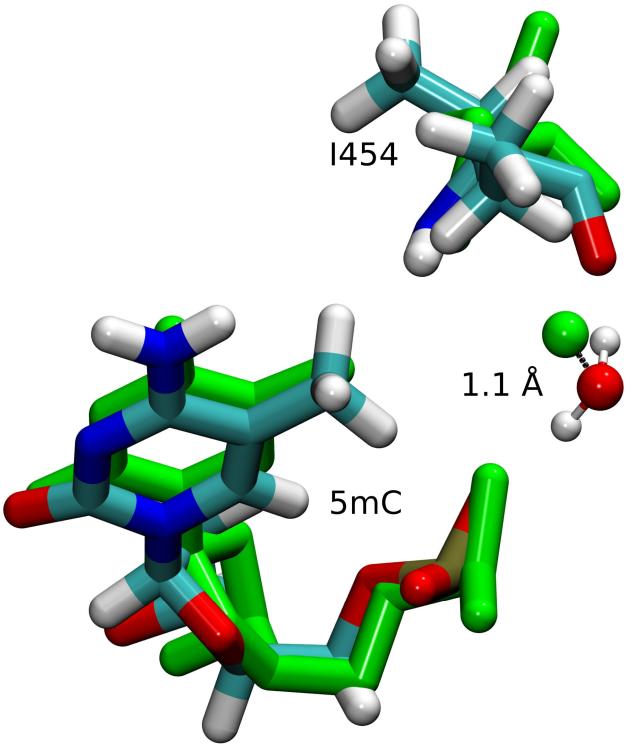

Supplementary Figure S5. Superposition of the equilibrated 5mC structure after simulation (atom-name specific coloring) and the crystal structure (PDB-ID:3fde [2], green). The 5mC nucleotide, the residue I454 of the SRA binding pocket and the conserved water molecule are shown. Note that the distance between the oxygen atoms of the conserved water molecules in the two structures is only 1.1 Å.

2. Hashimoto H, Horton JR, Zhang X, Bostick M, Jacobsen SE, et al. (2008) The SRA domain of UHRF1 flips 5-methylcytosine out of the DNA helix. Nature 455: 826-829.
