# Supplementary material for: Recognition of 5-Hydroxymethylcytosine by the Uhrf1 SRA Domain
Source: PLoS One. 2011 Jun 22;6(6):e21306. doi: 10.1371/journal.pone.0021306 (PMC3120858; doi:10.1371/journal.pone.0021306)
Supplement: Figure S6 — Molecular dynamics simulations of the Uhrf1 SRA domain in complex with 5mC (A) and 5hmC (B) containing DNA in 0.5 M NaCl. Hydrogen bond occurrences during the simulation of the SRA:DNA complex using a concentration of 0.5 M NaCl. (PDF) [file pone.0021306.s006.pdf]

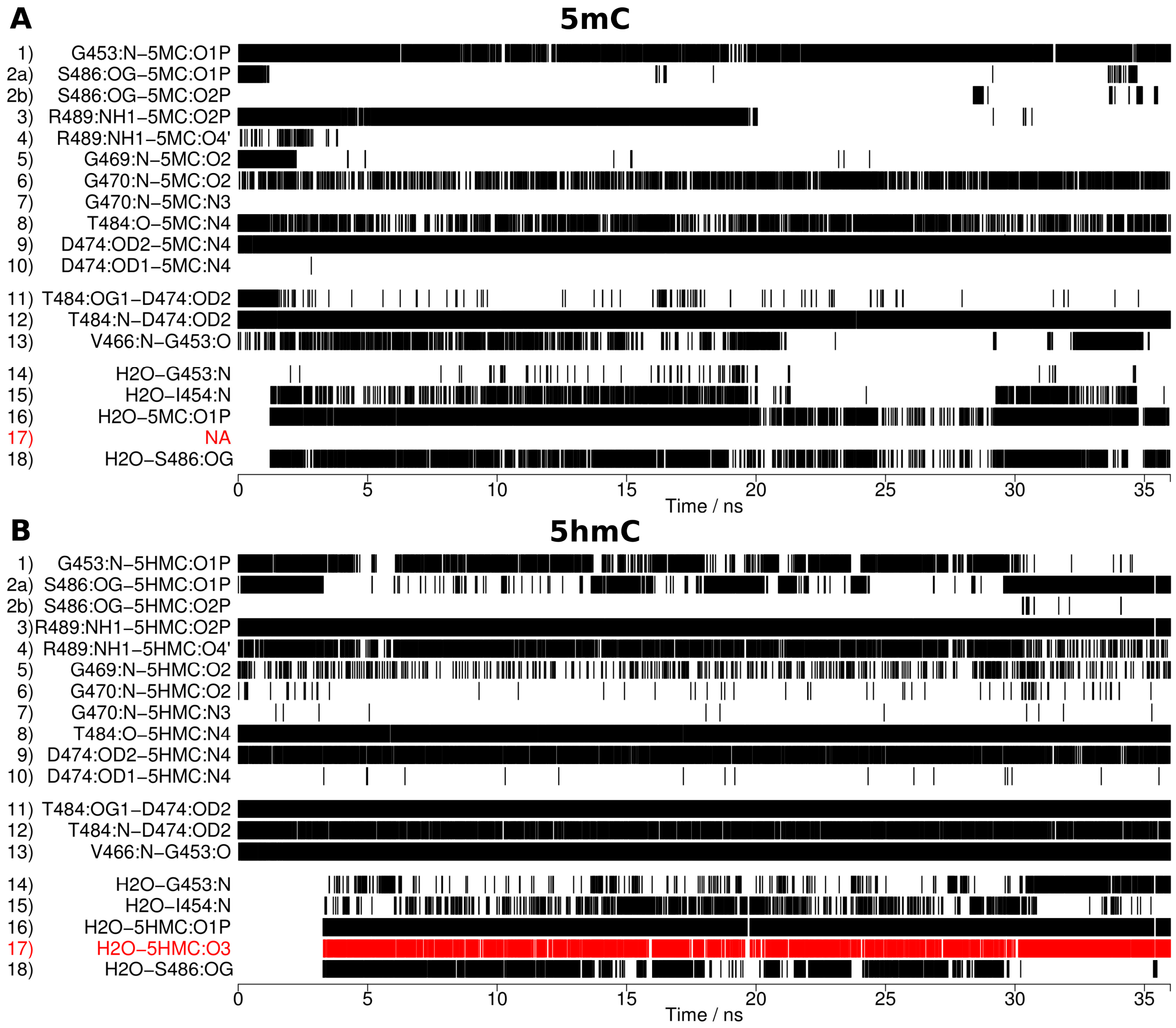

Supplementary Figure S6. Molecular dynamics simulations of the Uhrf1 SRA domain in complex with 5mC (A) and 5hmC (B) containing DNA in 0.5 M NaCl. Hydrogen bond occurrences during the simulation of the SRA:DNA complex using a concentration of 0.5 M NaCl.
