# Supplementary material for: Recognition of 5-Hydroxymethylcytosine by the Uhrf1 SRA Domain
Source: PLoS One. 2011 Jun 22;6(6):e21306. doi: 10.1371/journal.pone.0021306 (PMC3120858; doi:10.1371/journal.pone.0021306)
Supplement: Table S1 — Sequences of DNA oligonucleotides used for preparation of double stranded fluorescent DNA substrates. M: 5-methylcytosine. X: 5-hydroxymethylcytosine. (PDF) [file pone.0021306.s007.pdf]

**Supplementary Table S1. Sequences of DNA oligonucleotides used for preparation of double stranded fluorescent DNA substrates.**

M: 5-methylcytosine. X: 5-hydroxymethylcytosine.

| Name | Sequence |
|---|---|
| **CGup** | 5′- CTCAACAACTAACTACCATCCGGACCAGAAGAGTCATCATGG -3′ |
| **MGup** | 5′- CTCAACAACTAACTACCATCMGGACCAGAAGAGTCATCATGG -3′ |
| **hmCGup** | 5'- CTCAACAACTAACTACCATCXGGACCAGAAGAGTCATCATGG -3' |
| **noCGup** | 5′- CTCAACAACTAACTACCATCTGGACCAGAAGAGTCATCATGG -3′ |
| **um550** | 5′- ATTO550-CCATGATGACTCTTCTGGTCCGGATGGTAGTTAGTTGTTGAG -3′ |
| **um590** | 5′- ATTO590-CCATGATGACTCTTCTGGTCCGGATGGTAGTTAGTTGTTGAG -3′ |
| **um647N** | 5′- ATTO647N-CCATGATGACTCTTCTGGTCCGGATGGTAGTTAGTTGTTGAG -3′ |
| **um700** | 5′- ATTO700-CCATGATGACTCTTCTGGTCCGGATGGTAGTTAGTTGTTGAG -3′ |
| **mC700** | 5'- ATTO700-CCATGATGACTCTTCTGGTCMGGATGGTAGTTAGTTGTTGAG -3' |
| **hmC550** | 5'- ATTO550-CCATGATGACTCTTCTGGTCXGGATGGTAGTTAGTTGTTGAG -3' |
| **550-Fill-In** | 5′- ATTO550-CCATGATGACTCTTCTGGTC -3′ |
