# Supplementary material for: Recognition of 5-Hydroxymethylcytosine by the Uhrf1 SRA Domain
Source: PLoS One. 2011 Jun 22;6(6):e21306. doi: 10.1371/journal.pone.0021306 (PMC3120858; doi:10.1371/journal.pone.0021306)
Supplement: Table S2 — DNA substrates used for the DNA binding assays. (PDF) [file pone.0021306.s008.pdf]

Supplementary Table S2. DNA substrates used for the DNA binding assays.

Name	CpG site	Label	Oligo I	Oligo II	Purification grade and use
HMB550	hemimethylated	ATTO550	MGup	um550	· hybridization of HPLC-purified oligos<br>· gel-purification<br>· used for data in figure 2 and supplementary figure 1
HMB700	hemimethylated	ATTO700	MGup	um700	
HhMB700	hemihydroxymethylated	ATTO700	hmCGup	um700	
FMB700	fully methylated	ATTO700	MGup	mC700	
FhMB550	fully hydroxymethylated	ATTO550	hmCGup	hmC550	
noCG550	no CpG site	ATTO550	noCGup	550-Fill-In	· primer extension for noCG550<br>· hybridization of HPLC-purified oligos for HMB substrates<br>· gel-purification<br>· used for data in supplementary figure 2A
HMB550	hemimethylated	ATTO550	MGup	um550	
HMB647N	hemimethylated	ATTO647N	MGup	um647N	
UMB550	unmethylated	ATTO550	CGup	um550	· hybridization of HPLC-purified oligos<br>· used for data in supplementary figure 2B, n=2
UMB590	unmethylated	ATTO590	CGup	um590	
HMB590	hemimethylated	ATTO590	MGup	um590	
UMB647N	unmethylated	ATTO647N	CGup	um647N	· hybridization of PAGE-purified oligos<br>· used for data in supplementary figure 2B, n=1
UMB700	unmethylated	ATTO700	CGup	um700	
HMB700	hemimethylated	ATTO700	MGup	um700	
