# Supplementary material for: Recognition of 5-Hydroxymethylcytosine by the Uhrf1 SRA Domain
Source: PLoS One. 2011 Jun 22;6(6):e21306. doi: 10.1371/journal.pone.0021306 (PMC3120858; doi:10.1371/journal.pone.0021306)
Supplement: Table S3 — Residue Topology File and parameters used for the 5hmC residue during the simulations. (PDF) [file pone.0021306.s009.pdf]

Supplementary Table S3. Residue Topology File and parameters used for the 5hmC residue during the simulations.

```
================================================================================
TOPOLOGY (based on 5mC topology from patches: PRES 5MC2 and PRES DEO1)
================================================================================
! 5-hydroxy-methyl cytosine
RESI 5HMC        -1.00  !
ATOM P    P       1.50  !
ATOM O1P  ON3    -0.78  !                       H42  H41
ATOM O2P  ON3    -0.78  !                         \  /
ATOM O5'  ON2    -0.57  !                          N4
ATOM C5'  CN8B   -0.08  !                          |
ATOM H5'  HN8     0.09  !               H3-O3      C4
ATOM H5'' HN8     0.09  !                   \     /  \\
GROUP                   !              H5M2 -C5M-C5   N3
ATOM C4'  CN7     0.16  !                   /    ||   |
ATOM H4'  HN7     0.09  !               H5M1  H6-C6   C2
ATOM O4'  ON6    -0.50  !                         \  / \\
ATOM C1'  CN7B    0.16  !                          N1   O2
ATOM H1'  HN7     0.09  !                           \
GROUP                   !                            \
ATOM N1   NN2    -0.13  !                             \
ATOM C6   CN3     0.05  !         O1P    H5' H4'  O4'  \
ATOM H6   HN3     0.17  !          |      |    \ /   \  \
ATOM C5   CN3D   -0.11  !         -P-O5'-C5'---C4'    C1'
ATOM C5M  CN9     0.10  !          |      |     \     / \
ATOM H5M1 HN9     0.09  !         O2P    H5''   C3'--C2' H1'
ATOM H5M2 HN9     0.09  !                      / \   / \
ATOM O3   OH1    -0.66  !                   O3' H3' H2' H2''
ATOM H3   H       0.43  !                    |
ATOM C2   CN1     0.52  !                    |
ATOM O2   ON1C   -0.49  !
ATOM N3   NN3    -0.66  !
ATOM C4   CN2     0.65  !
ATOM N4   NN1    -0.75  !
ATOM H41  HN1     0.37
ATOM H42  HN1     0.33
GROUP
ATOM C2'  CN8    -0.18  !
ATOM H2'' HN8     0.09  !
ATOM H2'  HN8     0.09  !
GROUP
ATOM C3'  CN7     0.01
ATOM H3'  HN7     0.09
ATOM O3'  ON2    -0.57
BOND P    O1P       P    O2P       P     O5'
BOND O5'  C5'       C5'  C4'       C4'  O4'      C4'  C3'    O4'  C1'
BOND C1'  N1        C1'  C2'       N1   C2       N1   C6
BOND C2   N3        C4   N4        N4   H41      N4   H42
BOND C4   C5        C2'  C3'       C3'  O3'      O3'  +P
BOND C1'  H1'       C2'  H2''      C2'  H2'      C3'  H3'    C4'  H4'    C5'  H5'
BOND C5'  H5''      C6   H6
BOND C5   C5M  C5M  H5M1  C5M H5M2  C5M  O3  O3 H3
ANGL C4   C5   C5M   C6   C5   C5M
ANGL C5   C5M  H5M1  C5   C5M  H5M2  C5   C5M  O3  C5M O3 H3
ANGL H5M1 C5M  H5M2  H5M1 C5M  O3    H5M2 C5M  O3
DIHE C5M  C5   C4   N3   C5M  C5   C4   N4
DIHE C5M  C5   C6   H6   C5M  C5   C6   N1
DIHE H5M1 C5M  C5   C4   H5M1 C5M  C5   C6
DIHE H5M2 C5M  C5   C4   H5M2 C5M  C5   C6
DIHE O3   C5M  C5   C4   O3   C5M  C5   C6
DIHE H3   O3   C5M  C5 H3   O3   C5M  H5M2
DIHE H3   O3   C5M  H5M1
DOUBLE   C2   O2    C5   C6         N3   C4
IMPR C2   N1   N3   O2        C4   N3   C5   N4
IMPR N4   C4   H41  H42
DONO H42  N4
DONO H41  N4
DONO H3   O3
ACCE O2   C2
ACCE N3
ACCE O1P  P
ACCE O2P  P
ACCE O3'
ACCE O4'
```

```
ACCE O5'
ACCE O3

BILD -O3' P    O5'  C5'    1.6001  101.45  -46.90  119.00   1.4401 !alpha
BILD -O3' O5'  *P   O1P    1.6001  101.45 -115.82  109.74   1.4802
BILD -O3' O5'  *P   O2P    1.6001  101.45  115.90  109.80   1.4801
BILD  P   O5'  C5'  C4'    1.5996  119.00 -146.00  110.04   1.5160 !beta
BILD O5'  C5'  C4'  C3'    1.4401  108.83   60.00  116.10   1.5284 !gamma
BILD C5'  C4'  C3'  O3'    1.5160  116.10  140.00  115.12   1.4212 !delta
BILD C4'  C3'  O3'  +P     1.5284  111.92  155.00  119.05   1.6001 !epsilon
BILD C3'  O3'  +P   +O5'   1.4212  119.05  -95.20  101.45   1.5996 !zeta
BILD O4'  C3'  *C4' C5'    1.4572  104.06 -120.04  116.10   1.5160
BILD C2'  C4'  *C3' O3'    1.5284  100.16 -124.08  115.12   1.4212
BILD C4'  C3'  C2'  C1'    1.5284  100.16  -30.00  102.04   1.5251
BILD C3'  C2'  C1'  N1     1.5284  101.97  147.89  113.71   1.4896
BILD O4'  C1'  N1   C2     1.5251  113.71  -97.2   125.59   1.3783 !chi
BILD C1'  C2   *N1  C6     1.4896  117.79 -180.00  120.6    1.364
BILD C2   N1   C6   C5     1.399   120.6      0.0  121.0    1.337
BILD C6   N1   C2   N3     1.364   120.6      0.0  118.9    1.356
BILD N1   N3   *C2  O2     1.399   118.9    180.0  121.9    1.237
BILD N1   C2   N3   C4     1.399   118.9      0.0  120.0    1.334
BILD C5   N3   *C4  N4     1.426   121.8    180.00  118.9   1.337
BILD N3   C4   N4   H41    1.337   117.9      0.00  118.9   1.01
BILD H41  C4   *N4  H42    1.01    118.9    180.00  120.7   1.01
BILD N1   C5   *C6  H6     0.0       0.0    180.0     0.0   0.0
BILD C1'  C3'  *C2' H2'    1.5284  102.04 -114.67  110.81   1.01
BILD O4'  C2'  *C1' H1'    0.0       0.0  -115.0     0.0    0.0
BILD C1'  C3'  *C2' H2''   0.0       0.0   115.0     0.0    0.0
BILD C1'  C3'  *C2' H2'    0.0       0.0  -115.0     0.0    0.0
BILD C2'  C4'  *C3' H3'    0.0       0.0   115.0     0.0    0.0
BILD C3'  O4'  *C4' H4'    0.0       0.0  -115.0     0.0    0.0
BILD C4'  O5'  *C5' H5'    0.0       0.0  -115.0     0.0    0.0
BILD C4'  O5'  *C5' H5''   0.0       0.0   115.0     0.0    0.0
BILD C6   C4   *C5  C5M    0.0       0.0   180.0     0.0    0.0
BILD C4   C5   C5M  H5M1   0.0       0.0   180.0     0.0    0.0
BILD C5   H5M1 *C5M H5M2   0.0       0.0  -115.0     0.0    0.0
BILD H5M1 H5M2 *C5M O3     0.0       0.0   115.0     0.0    0.0
BILD C4   C5   C5M  O3     0.0       0.0    60.0     0.0    0.0
BILD C5   C5M  O3   H3     0.0       0.0   180.0     0.0    0.0
================================================================================

================================================================================
FORCEFIELD PARAMETERS:
================================================================================
...
BONDS
...
!added for 5HMC TU_TCB TH und ISA
OH1  CN9   428.000     1.4200 ! ACC. TO OH1-CT3
...
ANGLES
...
!added for 5HMC TU_TCB TH und ISA
OH1  CN9  CN3D   75.700   110.1000 !ACC. TO  OH1-CT2-CT2
OH1  CN9  HN9    45.900   108.8900 !ACC. TO  OH1-CT3-HA
H    OH1  CN9    57.500   106.0000 !ACC. TO  H-OH1-CT2
...
DIHEDRALS
...
!added for 5HMC TU_TCB TH und ISA
H    OH1  CN9  CN3D     1.3000  1     0.00 !ACC. TO  H-OH1-CT2-CT2
H    OH1  CN9  CN3D     0.3000  2     0.00 !ACC. TO  H-OH1-CT2-CT2
H    OH1  CN9  CN3D     0.4200  3     0.00 !ACC. TO  H-OH1-CT2-CT2
CN3  CN3D CN9  OH1      0.0     3     0.0  !ACC. TO  CN3-CN3D-CN9-HN9
CN2  CN3D CN9  OH1      0.35    3     0.0  !ACC. TO  CN3-CN3D-CN9-HN9
HN9  CN9  OH1  H        0.1400  3     0.00 !ACC. TO  X-CT2-OH1-X
...
IMPROPER
...
```
