# Supplementary material for: Recognition of 5-Hydroxymethylcytosine by the Uhrf1 SRA Domain
Source: PLoS One. 2011 Jun 22;6(6):e21306. doi: 10.1371/journal.pone.0021306 (PMC3120858; doi:10.1371/journal.pone.0021306)
Supplement: File S1 — Combined supporting figures and tables. (PDF) [file pone.0021306.s010.pdf]

Supplementary Material

Recognition of 5-hydroxymethylcytosine by the Uhrf1 SRA domain

Carina Frauer, Thomas Hoffmann, Sebastian Bultmann, Valentina Casa, M. Cristina Cardoso, Iris Antes and Heinrich Leonhardt

Supplementary Tables

Supplementary Table S1. Sequences of DNA oligonucleotides used for preparation of double stranded fluorescent DNA substrates.

M: 5-methylcytosine. X: 5-hydroxymethylcytosine.

Name	Sequence
**CGup**	5′- CTCAACAACTAACTACCATCCGGACCAGAAGAGTCATCATGG -3′
**MGup**	5′- CTCAACAACTAACTACCATCMGGACCAGAAGAGTCATCATGG -3′
**hmCGup**	5'- CTCAACAACTAACTACCATCXGGACCAGAAGAGTCATCATGG -3'
**noCGup**	5′- CTCAACAACTAACTACCATCTGGACCAGAAGAGTCATCATGG -3′
**um550**	5′- ATTO550-CCATGATGACTCTTCTGGTCCGGATGGTAGTTAGTTGTTGAG -3′
**um590**	5′- ATTO590-CCATGATGACTCTTCTGGTCCGGATGGTAGTTAGTTGTTGAG -3′
**um647N**	5′- ATTO647N-CCATGATGACTCTTCTGGTCCGGATGGTAGTTAGTTGTTGAG -3′
**um700**	5′- ATTO700-CCATGATGACTCTTCTGGTCCGGATGGTAGTTAGTTGTTGAG -3′
**mC700**	5'- ATTO700-CCATGATGACTCTTCTGGTCMGGATGGTAGTTAGTTGTTGAG -3'
**hmC550**	5'- ATTO550-CCATGATGACTCTTCTGGTCXGGATGGTAGTTAGTTGTTGAG -3'
**550-Fill-In**	5′- ATTO550-CCATGATGACTCTTCTGGTC -3′

Supplementary Table S2. DNA substrates used for the DNA binding assays.

Name	CpG site	Label	Oligo I	Oligo II	Purification grade and use
HMB550	hemimethylated	ATTO550	MGup	um550	· hybridization of HPLC-purified oligos
HMB700	hemimethylated	ATTO700	MGup	um700	· gel-purification
HhMB700	hemihydroxymethylated	ATTO700	hmCGup	um700	· used for data in figure 2 and supplementary figure 1
FMB700	fully methylated	ATTO700	MGup	mC700	
FhMB550	fully hydroxymethylated	ATTO550	hmCGup	hmC550	
noCG550	no CpG site	ATTO550	noCGup	550-Fill-In	· primer extension for noCG550
HMB550	hemimethylated	ATTO550	MGup	um550	· hybridization of HPLC-purified oligos for HMB substrates
HMB647N	hemimethylated	ATTO647N	MGup	um647N	· gel-purification
					· used for data in supplementary figure 2A
UMB550	unmethylated	ATTO550	CGup	um550	· hybridization of HPLC-purified oligos
UMB590	unmethylated	ATTO590	CGup	um590	· used for data in supplementary figure 2B, n=2
HMB590	hemimethylated	ATTO590	MGup	um590	
UMB647N	unmethylated	ATTO647N	CGup	um647N	· hybridization of PAGE-purified oligos
UMB700	unmethylated	ATTO700	CGup	um700	· used for data in supplementary figure 2B, n=1
HMB700	hemimethylated	ATTO700	MGup	um700	

Supplementary Table S3. Residue Topology File and parameters used for the 5hmC residue during the simulations.

```
===============================================================================
TOPOLOGY (based on 5mC topology from patches: PRES 5MC2 and PRES DEO1)
===============================================================================
! 5-hydroxy-methyl cytosine
RESI 5HMC        -1.00  !
ATOM P    P       1.50  !
ATOM O1P  ON3    -0.78  !                      H42  H41
ATOM O2P  ON3    -0.78  !                        \  /
ATOM O5'  ON2    -0.57  !                         N4
ATOM C5'  CN8B   -0.08  !                         |
ATOM H5'  HN8     0.09  !             H3-O3       C4
ATOM H5'' HN8     0.09  !                 \     /  \\
GROUP                   !            H5M2 -C5M-C5   N3
ATOM C4'  CN7     0.16  !                 /    ||   |
ATOM H4'  HN7     0.09  !             H5M1  H6-C6   C2
ATOM O4'  ON6    -0.50  !                        \  / \\
ATOM C1'  CN7B    0.16  !                         N1   O2
ATOM H1'  HN7     0.09  !                          \
GROUP                   !                           \
ATOM N1   NN2    -0.13  !                            \
ATOM C6   CN3     0.05  !        O1P    H5' H4'  O4'  \
ATOM H6   HN3     0.17  !         |      |    \ /   \  \
ATOM C5   CN3D   -0.11  !        -P-O5'-C5'---C4'    C1'
ATOM C5M  CN9     0.10  !         |      |     \     / \
ATOM H5M1 HN9     0.09  !        O2P    H5''   C3'--C2' H1'
ATOM H5M2 HN9     0.09  !                     / \   / \
ATOM O3   OH1    -0.66  !                  O3' H3' H2' H2''
ATOM H3   H       0.43  !                   |
ATOM C2   CN1     0.52  !                   |
ATOM O2   ON1C   -0.49  !
ATOM N3   NN3    -0.66  !
ATOM C4   CN2     0.65  !
ATOM N4   NN1    -0.75  !
ATOM H41  HN1     0.37
ATOM H42  HN1     0.33
GROUP
ATOM C2'  CN8    -0.18  !
ATOM H2'' HN8     0.09  !
ATOM H2'  HN8     0.09  !
GROUP
ATOM C3'  CN7     0.01
ATOM H3'  HN7     0.09
ATOM O3'  ON2    -0.57
BOND P    O1P       P    O2P       P     O5'
BOND O5'  C5'       C5'  C4'       C4'  O4'      C4'  C3'    O4'  C1'
BOND C1'  N1        C1'  C2'       N1   C2       N1   C6
BOND C2   N3        C4   N4        N4   H41      N4   H42
BOND C4   C5        C2'  C3'       C3'  O3'      O3'  +P
BOND C1'  H1'       C2'  H2''      C2'  H2'      C3'  H3'    C4'  H4'    C5'  H5'
BOND C5'  H5''      C6   H6
BOND C5   C5M  C5M  H5M1  C5M H5M2  C5M  O3  O3 H3
ANGL C4   C5   C5M   C6   C5   C5M
ANGL C5   C5M  H5M1  C5   C5M  H5M2  C5   C5M  O3  C5M O3 H3
ANGL H5M1 C5M  H5M2  H5M1 C5M  O3    H5M2 C5M  O3
DIHE C5M  C5   C4   N3   C5M  C5   C4   N4
DIHE C5M  C5   C6   H6   C5M  C5   C6   N1
DIHE H5M1 C5M  C5   C4   H5M1 C5M  C5   C6
DIHE H5M2 C5M  C5   C4   H5M2 C5M  C5   C6
DIHE O3   C5M  C5   C4   O3   C5M  C5   C6
DIHE H3   O3   C5M  C5 H3   O3   C5M  H5M2
DIHE H3   O3   C5M  H5M1
DOUBLE   C2   O2    C5   C6         N3   C4
IMPR C2   N1   N3   O2         C4   N3   C5   N4
IMPR N4   C4   H41  H42
DONO H42  N4
DONO H41  N4
DONO H3   O3
ACCE O2   C2
ACCE N3
ACCE O1P  P
ACCE O2P  P
```

```
ACCE O3'
ACCE O4'
ACCE O5'
ACCE O3

BILD -O3' P    O5'  C5'    1.6001  101.45  -46.90  119.00   1.4401 !alpha
BILD -O3' O5'  *P   O1P    1.6001  101.45 -115.82  109.74   1.4802
BILD -O3' O5'  *P   O2P    1.6001  101.45  115.90  109.80   1.4801
BILD  P   O5'  C5'  C4'    1.5996  119.00 -146.00  110.04   1.5160 !beta
BILD O5'  C5'  C4'  C3'    1.4401  108.83   60.00  116.10   1.5284 !gamma
BILD C5'  C4'  C3'  O3'    1.5160  116.10  140.00  115.12   1.4212 !delta
BILD C4'  C3'  O3'  +P     1.5284  111.92  155.00  119.05   1.6001 !epsilon
BILD C3'  O3'  +P   +O5'   1.4212  119.05  -95.20  101.45   1.5996 !zeta
BILD O4'  C3'  *C4' C5'    1.4572  104.06 -120.04  116.10   1.5160
BILD C2'  C4'  *C3' O3'    1.5284  100.16 -124.08  115.12   1.4212
BILD C4'  C3'  C2'  C1'    1.5284  100.16  -30.00  102.04   1.5251
BILD C3'  C2'  C1'  N1     1.5284  101.97  147.89  113.71   1.4896
BILD O4'  C1'  N1   C2     1.5251  113.71  -97.2   125.59   1.3783 !chi
BILD C1'  C2   *N1  C6     1.4896  117.79 -180.00  120.6    1.364
BILD C2   N1   C6   C5     1.399   120.6     0.0   121.0    1.337
BILD C6   N1   C2   N3     1.364   120.6     0.0   118.9    1.356
BILD N1   N3   *C2  O2     1.399   118.9   180.0   121.9    1.237
BILD N1   C2   N3   C4     1.399   118.9     0.0   120.0    1.334
BILD C5   N3   *C4  N4     1.426   121.8   180.00  118.9    1.337
BILD N3   C4   N4   H41    1.337   117.9     0.00  118.9    1.01
BILD H41  C4   *N4  H42    1.01    118.9   180.00  120.7    1.01
BILD N1   C5   *C6  H6     0.0       0.0   180.0     0.0    0.0
BILD C1'  C3'  *C2' H2'    1.5284  102.04 -114.67  110.81   1.01
BILD O4'  C2'  *C1' H1'    0.0       0.0  -115.0     0.0    0.0
BILD C1'  C3'  *C2' H2''   0.0       0.0   115.0     0.0    0.0
BILD C1'  C3'  *C2' H2'    0.0       0.0  -115.0     0.0    0.0
BILD C2'  C4'  *C3' H3'    0.0       0.0   115.0     0.0    0.0
BILD C3'  O4'  *C4' H4'    0.0       0.0  -115.0     0.0    0.0
BILD C4'  O5'  *C5' H5'    0.0       0.0  -115.0     0.0    0.0
BILD C4'  O5'  *C5' H5''   0.0       0.0   115.0     0.0    0.0
BILD C6   C4   *C5  C5M    0.0       0.0   180.0     0.0    0.0
BILD C4   C5   C5M  H5M1   0.0       0.0   180.0     0.0    0.0
BILD C5   H5M1 *C5M H5M2   0.0       0.0  -115.0     0.0    0.0
BILD H5M1 H5M2 *C5M O3     0.0       0.0   115.0     0.0    0.0
BILD C4   C5   C5M  O3     0.0       0.0    60.0     0.0    0.0
BILD C5   C5M  O3   H3     0.0       0.0   180.0     0.0    0.0
===============================================================================

===============================================================================
FORCEFIELD PARAMETERS:
===============================================================================
...
BONDS
...
!added for 5HMC TU_TCB TH und ISA
OH1  CN9   428.000     1.4200 ! ACC. TO OH1-CT3
...
ANGLES
...
!added for 5HMC TU_TCB TH und ISA
OH1  CN9  CN3D   75.700   110.1000 !ACC. TO  OH1-CT2-CT2
OH1  CN9  HN9    45.900   108.8900 !ACC. TO  OH1-CT3-HA
H    OH1  CN9    57.500   106.0000 !ACC. TO  H-OH1-CT2
...
DIHEDRALS
...
!added for 5HMC TU_TCB TH und ISA
H    OH1  CN9  CN3D     1.3000  1     0.00 !ACC. TO  H-OH1-CT2-CT2
H    OH1  CN9  CN3D     0.3000  2     0.00 !ACC. TO  H-OH1-CT2-CT2
H    OH1  CN9  CN3D     0.4200  3     0.00 !ACC. TO  H-OH1-CT2-CT2
CN3  CN3D CN9  OH1      0.0     3     0.0  !ACC. TO  CN3-CN3D-CN9-HN9
CN2  CN3D CN9  OH1      0.35    3     0.0  !ACC. TO  CN3-CN3D-CN9-HN9
HN9  CN9  OH1  H        0.1400  3     0.00 !ACC. TO  X-CT2-OH1-X
...
IMPROPER
...
```

Supplementary Figures

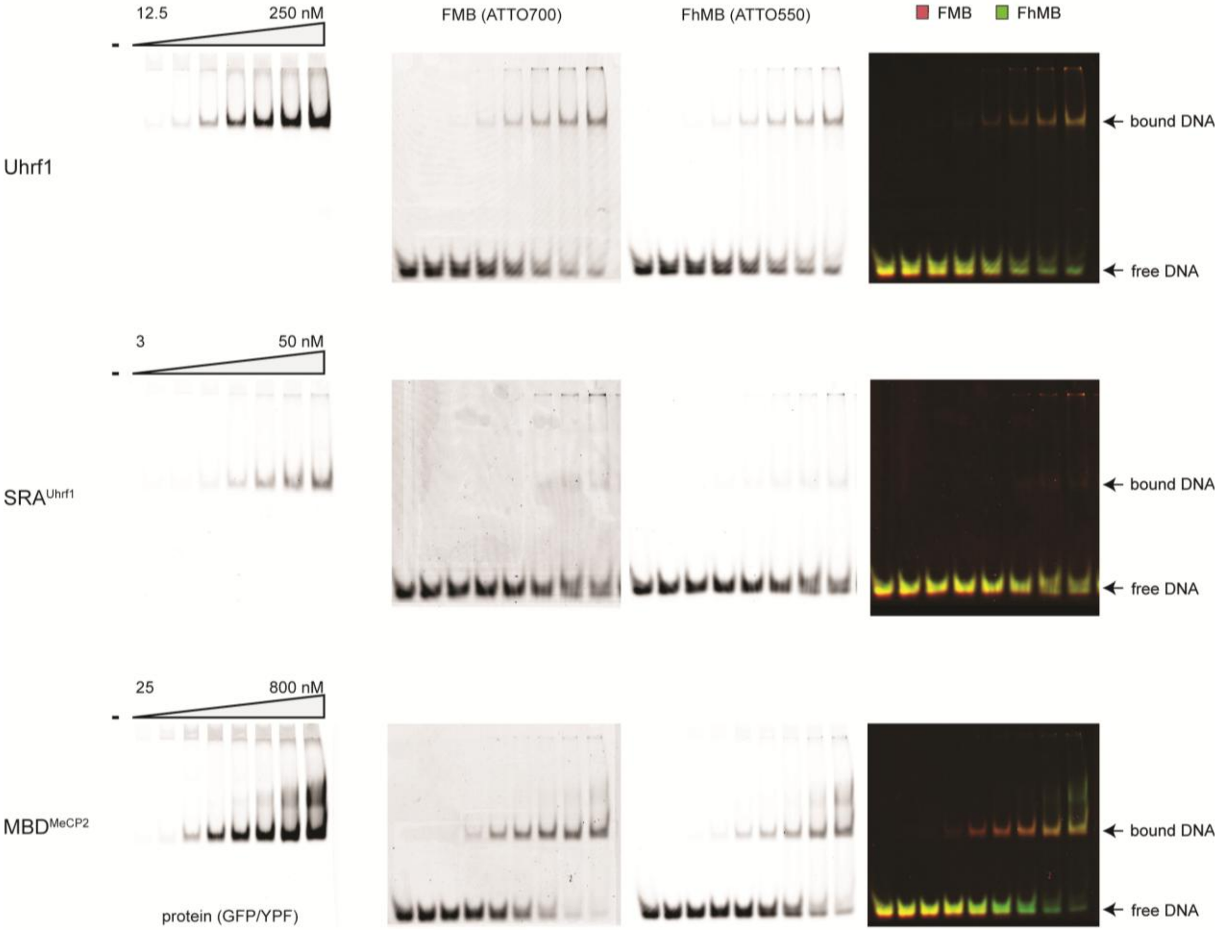

Supplementary Figure S1. Electrophoretic mobility shift assays with methylated and hydroxymethylated DNA substrates. Increasing amounts of Uhrf1, its SRA domain (SRA^{Uhrf1}) or the MBD domain of MeCP2 (MBD^{MeCP2}) were incubated with two differentially ATTO-labeled DNA substrates, which contain either one central fully methylated or fully hydroxymethylated CpG site (FMB-ATTO700 or FhMB-ATTO550, respectively), in direct competition. Samples were subjected to 6 % non-denaturing PAGE and analyzed with a Typhoon scanner (GE Healthcare). The first, second and third columns show the scans for GFP/YFP, ATTO700 and ATTO550 fluorescence, respectively. The overlay of the two ATTO channels is shown in the fourth column (FMB: red, FhMB:green).

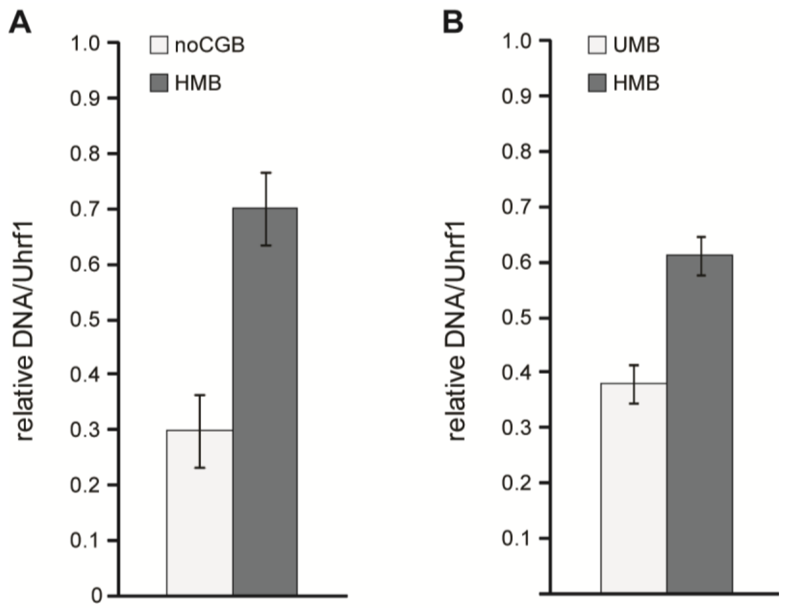

Supplementary Figure S2. DNA binding specificity of Uhrf1. Relative DNA/Uhrf1 ratios are shown for two differentially labeled fluorescent DNA substrates in direct competition. (A) Binding of Uhrf1 to DNA substrates containing no CpG site or one central hemimethylated CpG site (noCGB versus HMB, respectively). (B) Binding of Uhrf1 to DNA substrates containing one central un- or hemimethylated CpG site (UMB versus HMB, respectively). Results are shown as means of three independent experiments with standard deviation error bars. DNA substrates were prepared by hybridization as described in the main text, except for noCGB, which was prepared by primer extension as described previously [1]. See Supplementary Tables 1 and 2 for DNA oligonucleotide sequences and purification grade of the used substrates.

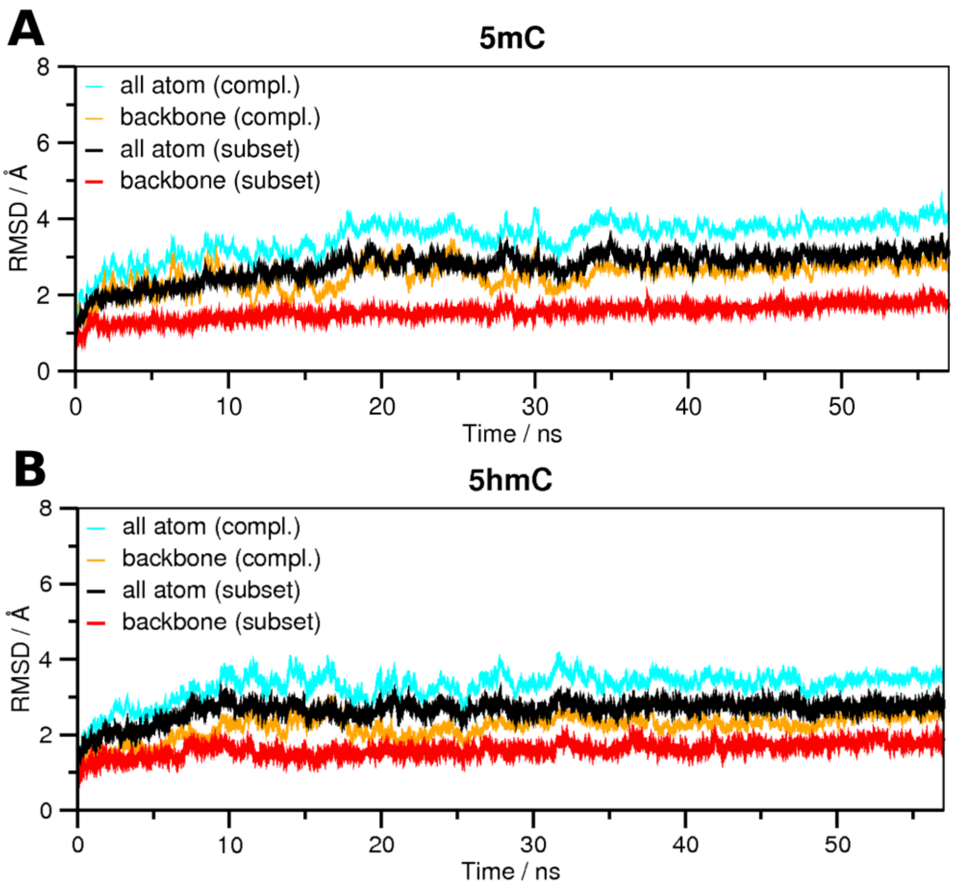

Supplementary Figure S3. Atom-positional root-mean-square deviation of the protein and DNA backbone atoms during the simulations. The terminal DNA and protein residues were excluded from the calculations in the "subset" sets (red and black lines).

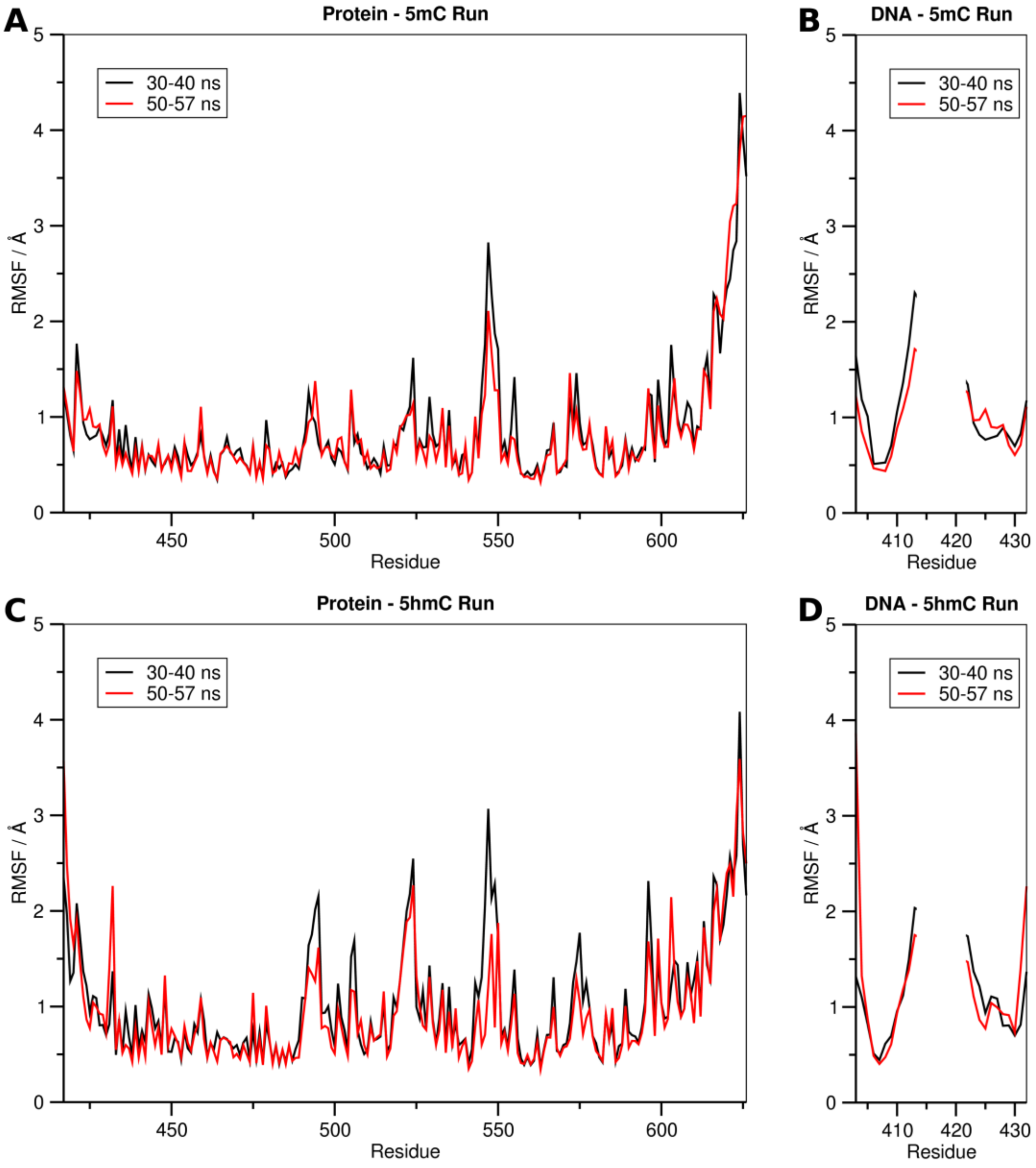

Supplementary Figure S4. Atom-positional root-mean-square fluctuations of the protein (A, C) and both DNA strands (B, D) during two simulation periods. Note that both structures show the same flexibility pattern during both simulation periods and are overall stable during both periods. This is in agreement with the RMSD data in Figure S3, which shows that equilibration is reached after 30 ns of simulation time.

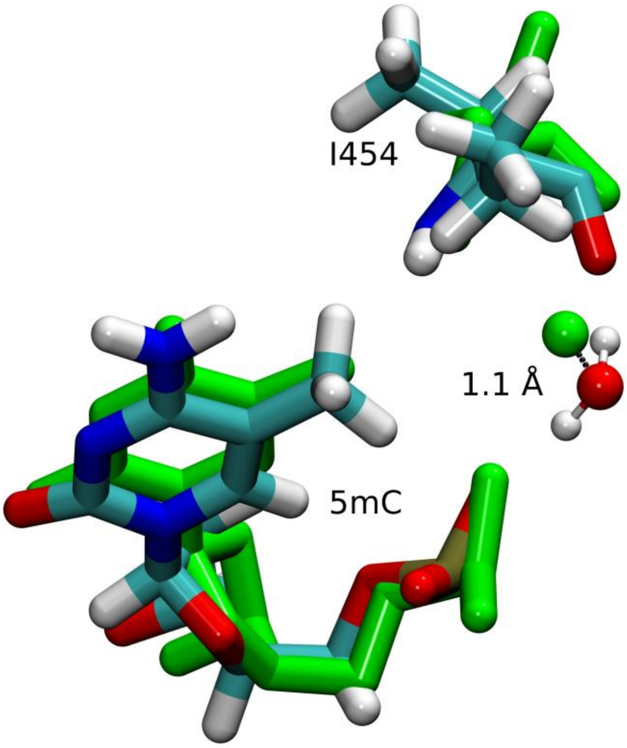

Supplementary Figure S5. Superposition of the equilibrated 5mC structure after simulation (atom-name specific coloring) and the crystal structure (PDB-ID:3fde [2], green). The 5mC nucleotide, the residue I454 of the SRA binding pocket and the conserved water molecule are shown. Note that the distance between the oxygen atoms of the conserved water molecules in the two structures is only 1.1 Å.

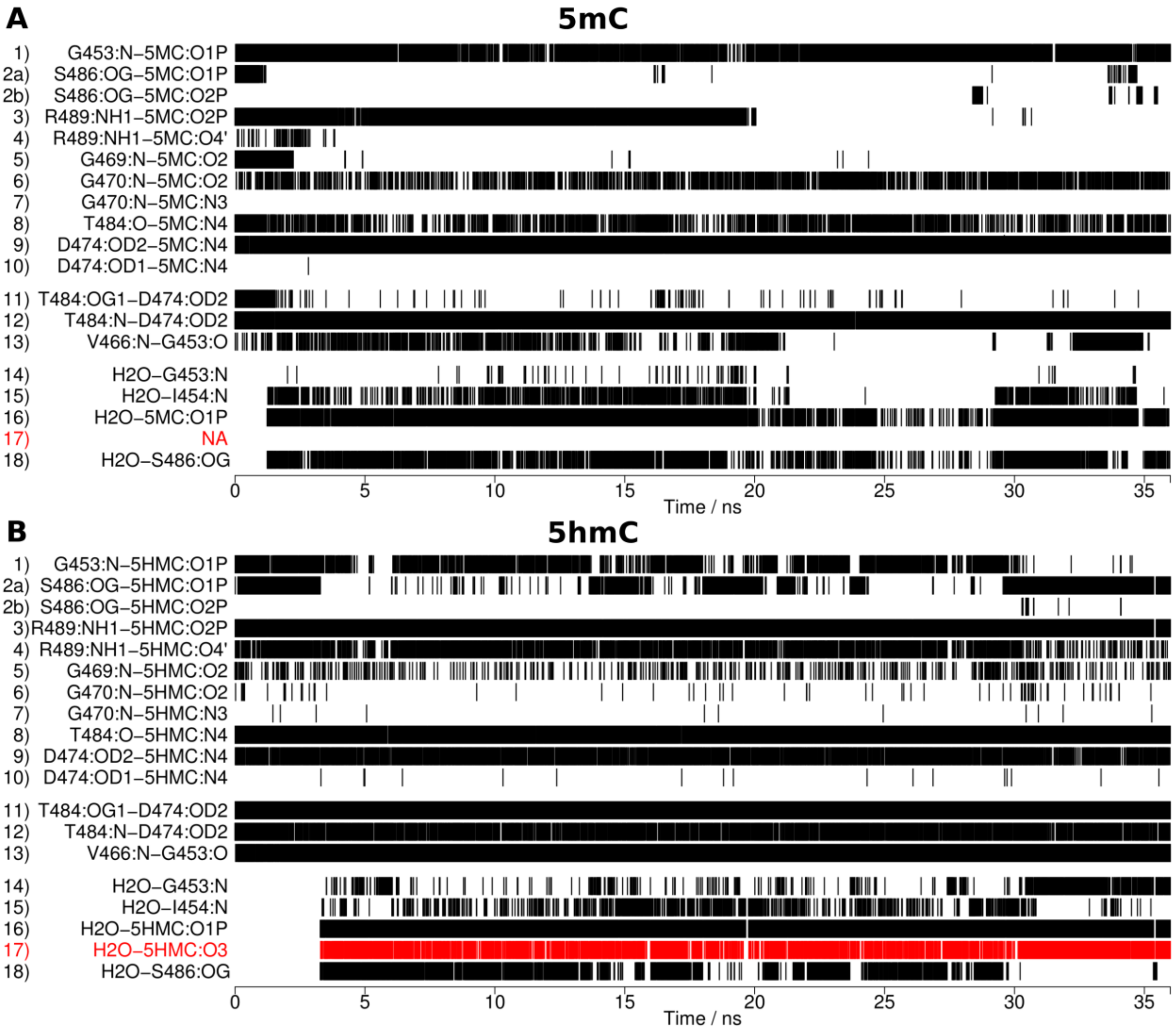

Supplementary Figure S6. Molecular dynamics simulations of the Uhrf1 SRA domain in complex with 5mC (A) and 5hmC (B) containing DNA in 0.5 M NaCl. Hydrogen bond occurrences during the simulation of the SRA:DNA complex using a concentration of 0.5 M NaCl.

Supplementary References

1. Frauer C, Leonhardt H (2009) A versatile non-radioactive assay for DNA methyltransferase activity and DNA binding. Nucleic Acids Res 37: e22.
2. Hashimoto H, Horton JR, Zhang X, Bostick M, Jacobsen SE, et al. (2008) The SRA domain of UHRF1 flips 5-methylcytosine out of the DNA helix. Nature 455: 826-829.
